# Pseudobulbar affect: clinical associations, social impact and quality of life implications - Lessons from PLS

**DOI:** 10.1007/s00415-025-12971-y

**Published:** 2025-03-12

**Authors:** Eoin Finegan, Jana Kleinerova, Orla Hardiman, Siobhan Hutchinson, Angela Garcia-Gallardo, Ee Ling Tan, Peter Bede

**Affiliations:** 1 Computational Neuroimaging Group (CNG), School of Medicine, Trinity College, Pearse Street, Dublin, Ireland; 2 Department of Neurology, St James’s Hospital, Dublin, Ireland

**Keywords:** Pseudobulbar affect, Pathological laughing and crying, Involuntary emotional expression disorder, Primary lateral sclerosis, Motor neuron disease, Neuropsychology

## Abstract

**Background:**

Pseudobulbar affect (PBA) is a well-recognised and troublesome clinical phenomenon in a range of neuroinflammatory, neoplastic, neurovascular and neurodegenerative conditions. It is often under-recognised in the community, frequently mistaken for psychiatric manifestations, appropriate pharmacological treatment is often delayed, and may result in a sense of embarrassment or lead to social isolation. Despite its considerable quality of life (QoL) implications and the challenges associated with its effective management, it is notoriously understudied.

**Methods:**

As the incidence of PBA is lower in non-motor neuron disease patient cohorts, and the social and QoL impact of PBA is not sufficiently recognised, a purpose-designed study was conducted in a Primary Lateral Sclerosis (PLS) cohort to assess the clinical correlates and social impact of PBA.

**Results:**

PBA was very strongly associated with pseudo-bulbar motor dysfunction. Dysphagia (OR 14, *P* = .005) and the presence of abnormal jaw jerk (OR 19.8, *P* < 0.001) greatly increased odds of PBA. There was no significant difference in the cognitive or behavioural profiles between those with PBA and those without it. Poorly controlled laughing (85%) was more prevalent than crying (69%) among PLS patients with PBA. No individual experienced PBA symptoms prior to the motor manifestations of PLS. Most patients were unaware that PBA was common in their neurological condition. The mean PBA Impact score was 5 (range 1–17) and correlated with CNS-LS crying subscores (r = .693, *p* = .006).

**Discussion:**

The severity of pseudobulbar affect correlates with motor manifestations of pseudobulbar palsy, a link supporting emerging imaging studies regarding bilateral corticobulbar tract degeneration as in important aetiological factor. The social and quality of life ramifications of pseudobulbar affect can be readily demonstrated by purpose-designed questionnaires.

**Conclusions:**

Despite sporadic reports, the clinical, social, caregiver burden and quality of life implications of pseudobulbar affect remain poorly characterised. The comprehensive evaluation of the clinical correlates of PBA helps to elucidate the underlying pathophysiology. Ultimately, the comprehensive assessment of both the aetiology and social impact of PBA helps to raise awareness of this entity, reduce misdiagnoses, enhance the early recognition of this phenomenon and encourage proactive pharmacological intervention.

## Background

The syndrome of pseudobulbar (or supra-nuclear) palsy, primarily characterised by spastic dysarthria and dysphagia, was described by Lepine in 1877 and was quickly recognised to occur in association with pathologies involving the bilateral cortico-bulbar tracts. The jaw-jerk reflex, an important clinical sign of pseudo-bulbar palsy was described soon afterwards by Lewis in 1882 [1]. The co-occurrence of pseudobulbar palsy and exaggerated emotional expression was described by Oppenheim and Siemering in 1886 who proposed disinhibition of brainstem centres as the probable mechanism—introducing the term, pseudobulbar affect (PBA) [2].

However, the association of PBA to bilateral corticobulbar pathology (pseudobulbar palsy) has been gradually de-emphasised as studies linked this phenomenon to widespread cortical, subcortical or cerebellar pathology and more recently to network disorders [3–11]. Innumerable, anatomically agnostic terms have been also used, focussing on phenomenology such as *pathological laughing and crying, involuntary emotional expression disorder, emotional lability, emotional incontinence* etc [12]. While this approach has advantages, there has been a surprising paucity of studies evaluating the clinical associations of PBA to test the traditional hypothesis and provide pathophysiological insights [13, 14]. While the co-occurrence of PBA and pseudobulbar motor features continues to be recognised, the strength of this association has rarely been systematically studied [15, 16]. Oppenheim, himself later alluded to the dilemma to what extent these symptoms should be attributed to “pure pseudo-bulbar paralysis” given the widespread cerebral pathology in studied cases [17]. Motor neuron diseases allow for systematic analysis of the clinical associations of PBA and explore the pathological underpinnings of PBA [5–7, 9, 13, 18, 19]. As the archetypical bilateral UMN disorder, primary lateral sclerosis (PLS) may hold several advantages to study these associations, particularly when considering patients with and without pseudobulbar affect [20]. Disease burden patterns in PLS [21, 22] are less anatomically heterogeneous than in other diseases associated with PBA, such as stroke [23], multiple sclerosis (MS) [24, 25], traumatic brain injury (TBI) [26], movement disorders (PD, MSA) [27, 28], dementia syndromes [29–32] or indeed amyotrophic lateral sclerosis (ALS) [18, 19, 33, 34]. In ALS, concurrent lower motor neuron degeneration may mask the presence or severity of UMN signs and, therefore, confound the interpretation [35]. In addition, the recognition of cognitive and behavioural deficits in PLS allows evaluation of non-motor, as well as motor associations of PBA, which may provide insights into the pathophysiology [22].

The commonly used patient screening tools for PBA have been demonstrated to possess high accuracy in diagnosis of PBA, with expert clinical diagnosis as the gold standard [36]. However, they do not adequately assess several important aspects of patient experience living with PBA symptoms. The clinical, social, caregiver burden and quality of life implications of PBA are poorly characterised in various patient cohorts due the paucity of prospective studies, despite the recognition of the considerable impact on social interactions, life-style, and misdiagnoses associated with PBA [19, 27, 28, 31]. Few studies have assessed patient awareness of PBA as a symptom of their neurological condition. Clinical trials in PBA have generally relied on PBA screening tools together with non-specific quality-of-life instruments as outcome measures [37, 38]. There is an unmet need to better understand the PBA features associated with greater symptom burden. Accordingly, the main objective of this study is to evaluate the motor and neuro-psychological associations of PBA in a cohort of patients with primary lateral sclerosis (PLS). In addition, our study aims to evaluate patient experiences of PBA and factors associated with greatest quality-of-life impact.

## Methods

Patients were recruited prospectively from a motor neuron disease clinic and diagnosed according to the PLS consensus diagnostic criteria [39]. The study was approved by the Ethics (Medical Research) Committee of Beaumont Hospital, Dublin, Ireland. Participants were stratified for PBA using the Center for Neurological Disease Lability Scale (CNS-LS), following systematic assessments of motor and neuropsychological function [36, 39]. Modifications to standardised scores were made where necessary, to exclude items that would be clearly confounding in the comparison of groups with and without PBA.

Clinical and demographic details including age, sex, symptom duration, and current anti-depressant use were systematically recorded. Functional status for bulbar and limb regions were evaluated using the ALSFRS-r [40]. Given the absence of lower motor neuron signs in PLS, patients with a bulbar sub-score < 12 were classified as having pseudobulbar functional impairment. ALSFRS-r bulbar sub-score items were used to classify patients according to the presence or absence of dysphagia, dysarthria and sialorrhoea. Assessment of upper motor neuron signs was performed following the Penn Upper Motor Neuron (UMN) burden score examination which outlines a standardised physical assessment of UMN signs in both bulbar and limb regions [41]. Composite scores were calculated for bulbar region to include positive Jaw-jerk, pout and palmo-mental reflex (UMN-bulbar; max score of 3) but excluding PBA. The gag reflex was not assessed. As part of the UMN burden score, the Babinski (plantar) reflexes was also assessed. Thirty-one of the 39 participating patients underwent genetic screening for ALS- and HSP-associated genetic variants as well as being screened for GGGGCC hexanucleotide repeat expansions in *C9orf72*. Methods for genetic screening have been described in our neuroimaging studies [22, 42, 43].

Mood and anxiety were assessed with the HADS questionnaire, modified to exclude items D8 (“slowed down”) and A11 (“on the move”), as has been implemented in previous ALS and PLS studies due to obvious confounding effect of motor disability. [44, 45] Cognitive screening was performed using ECAS [46].Patients were classified based on presence or absence of overall, as well as domain specific impairments based on published data [47]. Behavioural change was evaluated using the Frontal Systems Behavior scale (FrSBe), completed by the patients themselves and a family member/caregiver. Again, items clearly impacted by motor impairments or referring directly to emotional lability as an indicator of disinhibition, were excluded to reduce confounding error.

Patients classified with PBA were asked to complete a symptom experience form (Table 1) and a PBA impact questionnaire focusing on the psycho-social impact of the symptom (Table 2) to evaluate the association between PBA symptom burden and its impact. Finally, correlation analyses between clinical variables and CNS-LS score and sub-scores were performed.

**Table 1:**
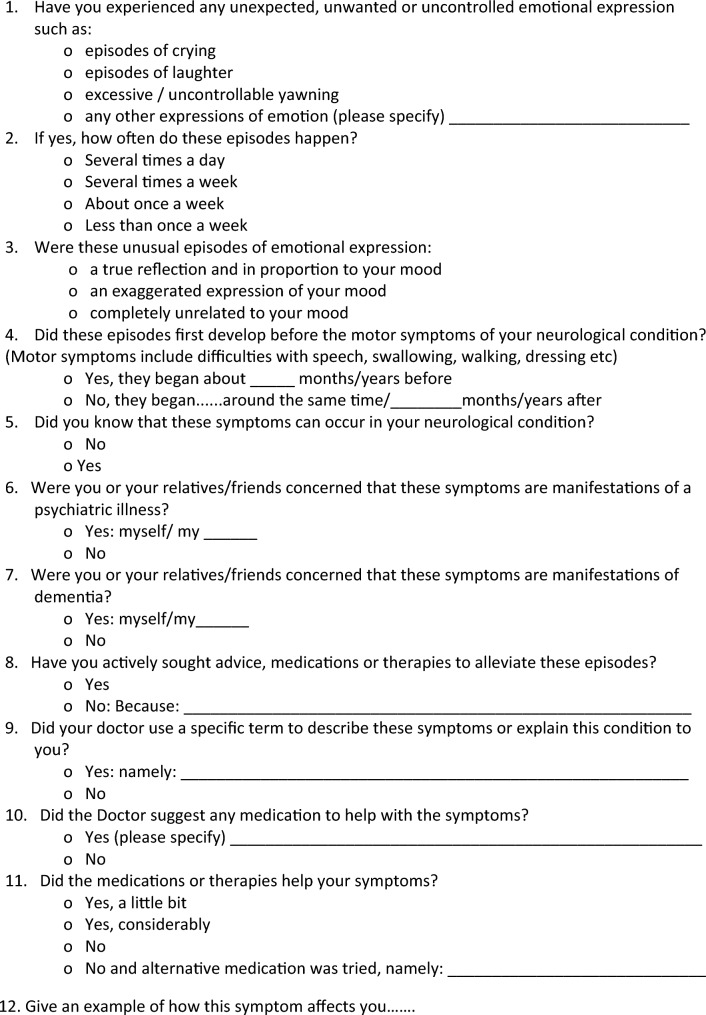
Symptom experience form

**Table 2.**
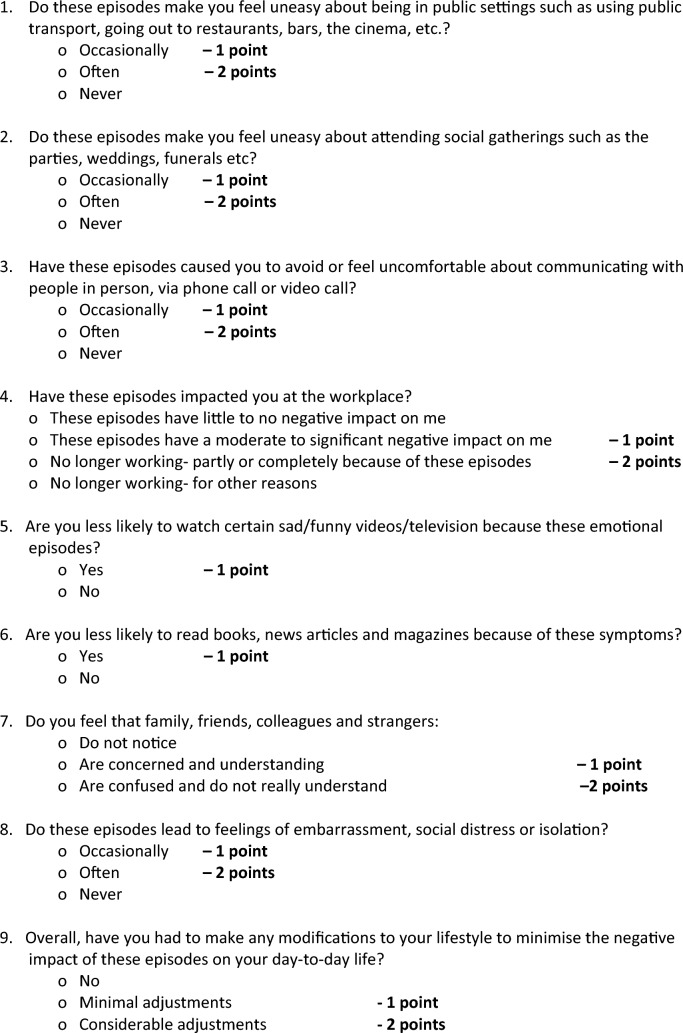
Pseudobulbar impact assessment tool

## Results

A total of 39 patients with PLS (61% male) participated in the study of whom 29 (74%) had pseudobulbar motor symptoms (dysarthria, dysphagia, sialorrhoea) at the time of assessment. Of these, dysarthria was the commonest manifestation (69%). All participants had limb symptoms and spasticity on formal neurological examination. A positive, brisk jaw-jerk was present in 29% the group. Fifteen patients were classified as PBA-positive (38%). Table 3 and Table 4 summarise the demographic and clinical characteristics of PBA-positive and PBA-negative groups. Patients who underwent genetic testing (*n* = 31) all tested negative for ALS- and HSP-associated genetic variants as well as GGGGCC hexanucleotide repeat expansions in *C9orf72*.

**Table 3:** The demographic and clinical profiles of study participants

	PBA + *n* = 15	PBA – *n* = 24	*p*-value
A: Demographic characteristics			
Age (years)	58.1	64.4	.053
PLS duration (months)	123.4	101.2	.346
Sex (male)	53%	67%	.405
Anti-depressant use (%)	33%	13%	.117
CNS-LS total	20.7	8.2	< .001
*B: Clinical Evaluation*			
Motor function			
ALSFRS-total	31	38	< .001
ALSFRS limbs	12.5	16.2	< .001
ALSFRS-bulbar	8	10.4	< .001
Neuropsychological screening			
HADS-Total*	7.5	4.9	.109
HADS- Dep*	2.2	1.6	.100
HADS-Anxiety*	5.3	2.9	.166
ECAS-impaired (Overall)	7%	22%	.224
Executive	7%	4%	.739
Language	27%	13%	.302
Memory	0%	13%	.162
Visuospatial	0%	4%	.437
FrSBe (Behaviour)**			
Disinhibition (relative)	19.7	17.1	.175
Disinhibition (self)	19.5	17.2	.133
Apathy (relative)	21.9	20.4	.302
Apathy (self)	24.5	20	.063
Executive (self)	31.6	30.5	.386
Executive (relative)	33.3	31.1	.362

^*^HADS excluded items: D8 (“slowed down”) and A11 (“on the move”, a

^**^FrSBe excluded items: Q1: speak only when spoken to. Q2: Emotional outburst without good reason. Q6: Laugh/Cry easily, Q8 Difficulty starting activity, Q12: Can’t sit still/hyperactive, Q17: Cannot do two things at once, multi-task Q29: slow moving, lacking energy, inactive

PBA was very strongly associated with pseudo-bulbar dysfunction. PBA was present in fifteen (52%) of patients with pseudo-bulbar dysfunction, but in no individual with normal bulbar function. Of the bulbar domains, the presence of dysphagia was associated with greatest odds of PBA (OR 14, *P* = 0.005). The presence of abnormal jaw jerk greatly increased odds of PBA (OR 19.8, *P* < 0.001). A Positive palmomental reflex and pout responses were common in both groups and were not significantly associated with PBA. Similarly, there was no difference in prevalence of bilateral Babinski sign.

There was no significant difference in cognitive impairment between the groups. There was no statistically significant difference in behaviour scores between study groups, including on assessment disinhibition. Similarly, PBA was not associated with higher scores on depression or anxiety scales.

**Fig. 1.**
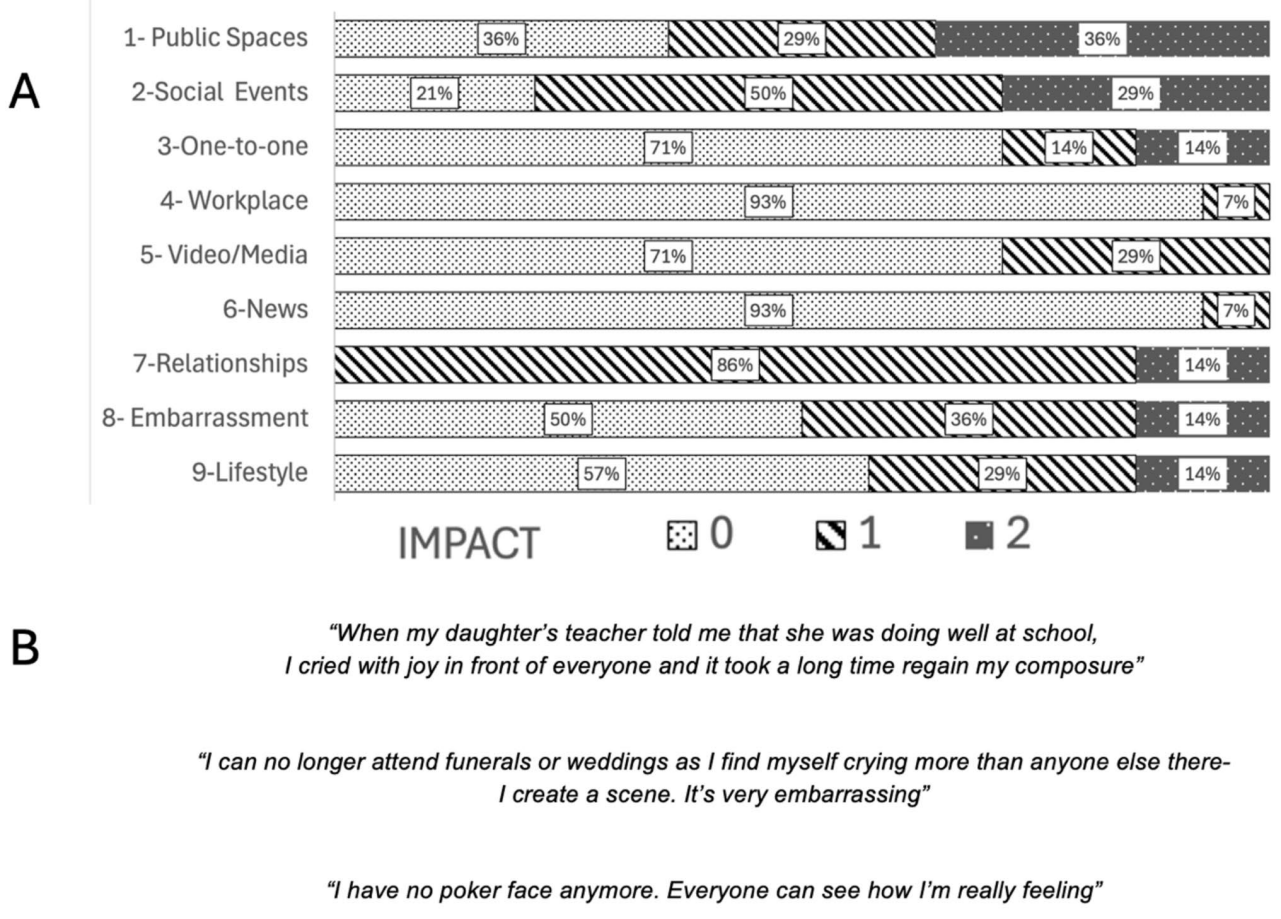
The social and quality of life impact of pseudobulbar affect

Fourteen patients (57% male) with PBA (CNS-LS mean 20, range 13–35) completed the symptom experience form and the PBA impact questionnaires. The results are presented in Fig. 1, and samples of patient accounts are highlighted in Fig. 1. Poorly controlled laughing was endorsed by 11/13 (85%) while crying was reported by 9/13 (69%). 6/14 (43%) reported frequency of several episodes daily. No individual reported that their PBA symptoms arose prior to the motor manifestations of the condition. All but one individual reported that episodes were an exaggerated reaction to a relevant emotional trigger and there was no case of episodes occurring without any trigger at all. One individual described an associated heightened emotional state when episodes occurred. Most (8/14) were not aware that PBA was common in their neurological condition. One individual felt it might be an indicator of a psychiatric condition. Just 3/14 (21%) patients were aware of any of the commonly used terms for the symptom; all of these using the term “lability” (or a derivation).

The mean PBA Impact score was 5/17 (range 1–17). Figure 1A illustrates the distribution of scores with higher score indicating greater impact. The PBA-IS was moderate-to-strongly correlated with CNS-LS crying (r = 0.693, *p* = 0.006), but not with laughing sub-scores (r = 0.390, *p* 0.168).

**Table 4:** The clinical characteristics of the study groups

	PBA +	PBA -	OR	*p*
Pseudo-bulbar palsy (dysfunction)	100%	58%	Infinite	**.004**
Dysphagia	93%	54%	14.0	**.005**
Dysarthria	93%	50%	11.8	**.010**
Sialorrhoea	80%	25%	12.0	** < .001**
UMN- Signs	79%	67%	1.8	.435
Jaw-Jerk	64%	8%	19.8	** < .001**
Pout	64%	46%	2.1	.272
Palmomental	50%	54%	0.8	.804
Babinski sign (Bilateral)	80%	75%	1.3	.718

Statistically significant differences are highlihgted in bold

## Discussion

Our study demonstrates that the severity of pseudobulbar affect is closely linked to the motor manifestations of pseudobulbar palsy. Contrary to previous studies [48], we found is no difference in the cognitive and behavioural profiles of those with PBA and those without it. These observations would implicate corticobulbar connectivity alterations as a primary driver of pseudobulbar affect, which is consistent with recent imaging papers evaluating the substrate of pseudobulbar affect [6, 7, 9, 13, 42]. Beyond the academic merits of clinical associations and putative pathomechanisms, this study demonstrates the “real-life” practical social and QoL impact of PBA. While the adverse implications of PBA are recognised by neurologists and there are anecdotal reports and case reports in the literature, there is limited awareness of this phenomenon amongst general physicians and other medical specialists. Rising awareness of PBA phenomenology (including yawning) among GPs, medical, psychiatric and nursing staff, is a meaningful pursuit and which may lead to the reduction of misdiagnosis, timely treatment and ultimately to improved quality of life of patient and their loved ones.

The consideration of PBA in relevant patient cohort has practical relevance, as patients may not exhibit typical features of PBA during short consultations. Neurologists should therefore specifically ask both the patient and their caregivers if they experience any of the cardinal features of PBA including excessive yawning. While unprovoked laughter or crying, or laughing or crying on minimal emotional stimuli are recognised features of PBA, excessive yawning is an overlooked facet of the PBA spectrum [49, 50]. In some situations, especially during the diagnostic work-up of incipient, early-phase neurodegenerative conditions, the detection of PBA may be diagnostic, or at least indicate cerebral or upper motor neuron involvement. In male patients for example, clinically presenting with tongue wasting and LMN-type bulbar dysfunction, SBMA or Kennedy’s disease may be a consideration [51, 52]. However, if features of PBA are reported or detected, ALS is a more likely diagnosis. The early accurate diagnosis of PLS is notoriously challenging as the core clinical features of PLS overlap with upper-motor neuron predominant ALS as well as HSP. [39, 53–56] Patients presenting with lower limb predominant spasticity with co-existing spinal canal stenosis may pose a diagnostic dilemma as to what extent are the spinal changes contributing to the symptoms confounding a potential neurodegenerative process such as PLS. These patients are typically monitored over time, unless there is evidence of PBA or a positive jaw jerk which would sway neurologist in favour of PLS. The early recognition of PBA has important clinical advantages. If helps to dissuade from misdiagnoses, reassure patients that it is a sequela of their primary disorder and not an additional psychiatric or psychological condition, and emphasise that a multitude of pharmacological intervention options exist with varying efficacy. There is often a sense of embarrassment amongst patients with severe PBA which may lead to social isolation, adding to the considerable psychological burden of a progressive neurological condition. Depending on the underlying condition, existing medications and the medical comorbidities (cardiac history, arrhythmias) pharmacological management options encompass citalopram [57], mirtazapine [58], fluoxetine [59], sertraline [60], amitriptyline [61] nortriptyline [38], and duloxetine [62], memantine [63] and more recently dextromethorphan/quinidine [37, 64, 65]. Accordingly, there are ample medications to consider and carefully titrate to control or decrease the symptoms associated with PBA.

Although, cognitive and behavioural changes are increasingly recognised in motor neuron diseases [66], PBA in this study, was not significantly associated with the presence of low mood, behavioural or cognitive impairment. This argues against PBA as a feature of a mood disorder or disinhibited behaviour. These findings are consistent with subjective accounts of patients’ experience of PBA as being generally mood congruent but disproportionate or exaggerated in their physical manifestation. We also note that none of the participating patients tested positive for *C9orf72.* GGGGCC hexanucleotide repeat expansion carrier status is often associated with marked frontotemporal and subcortical involvement and a range of neuropsychological manifestations [34, 67–69], but extensive extra-motor involvement is not unique to *C9orf72* hexanucleotide repeat expansion carriers [70].

In contrast, PBA was strongly associated with the presence of pseudobulbar symptoms and signs; most significantly with the presence of dysphagia, dysarthria and jaw-jerk hyper-reflexia. Remarkably, no individual without pseudobulbar symptoms was classified as having PBA. The presence of a hyper-active jaw-jerk reflex was associated with an almost 20-fold increase in the odds of being classified as having PBA. Previous authors have suggested that the presence of pathological increased brainstem reflexes may support the recognition of PBA although this is the first study to show an association with a pathologically increased jaw-jerk reflex [71]. A previous, retrospective study found a strong association between PBA and increased gag-reflex, which we did not test [35]. Interestingly that study also reported that dysarthria was present in all cases of PBA in that cohort.

Although other features of pseudobulbar palsy were present in all PLC patients in this study, this does not conclusively indicate PBA cannot occur in the absence of established pseudobulbar palsy. Neither does it imply that PBA may not be a presenting feature of pseudobulbar palsy in PBA, although, to our knowledge, this has not been described. However, lack of awareness of PBA, as was common in this cohort, may delay its presentation to medical attention prior to the onset of additional pseudobulbar symptoms such as dysarthria or dysphagia. Similarly, longitudinal studies are required to explore this chronological relationship between PBA and typical pseudobulbar motor features. Studies in ALS have reported high prevalence of PBA in bulbar-onset patients [16, 72].

These findings, taken together are supportive of Oppenheim’s classic hypothesis of disinhibited brainstem motor centres as underlying PBA and re-emphasise the central role of pseudobulbar motor dysfunction in this frequently distressing symptom. Surprisingly, although frequently challenged, the classical hypothesis has rarely been systematically tested in clinical studies. Revised models of PBA have drawn substantially from individual case reports of PBA in unexpected anatomical locations. Such reports, while instructive are likely influenced by reporting and publication bias of cases diverging from traditional model of cortico-bulbar dysfunction in PBA [73]. Case reports often infer clinico-anatomical correlation but do not include patients with anatomically similar lesions in whom PBA is not present, is yet to manifest or be recognised. Imaging studies have often relied on radiologically on overt lesions without knowledge of microstructural integrity of the cortico-bulbar tracts [4]. Although it is likely that the traditional model of brainstem motor disinhibition is itself inadequate to account for the spectrum of PBA presentations, larger case–control studies are required in cerebellar and other pathologies to better understand the role and relative contribution of mechanisms beyond cortico-bulbar pathology.

The results of this study do not directly address the hypothesised role of the cerebellum in modulating the expression of PBA [20]. Disruption of cortico-ponto-cerebellar pathways has been linked to PBA in previous imaging studies [13, 35]. Additionally, studies have consistently demonstrated that cerebellar degeneration is a feature of PLS [74, 75], although it is not established whether this is a primary pathological process or diaschisis related to the cerebral and spinal degenerative process [76]. However, it is clear from clinical studies in PLS that the pathological signature and clinical motor phenotype is dominated by upper motor neuron (cortico-bulbar and cortico-spinal) dysfunction and cerebellar dysfunction appears to be of secondary importance in the motor manifestations of PLS [22, 77]. Indeed, the diagnostic criteria do not require clinical cerebellar signs [39]. To our knowledge, no cohort study has demonstrated an association between clinical signs of cerebellar dysfunction and PBA in PLS. A comparison of clinical cerebellar dysfunction in affected and non-affected groups may be an instructive opportunity for future research.

The results of the symptom experience survey are broadly in agreement with previous studies [78, 79]. PBA should be regarded as a disorder of emotional expression, not of emotional content. In general, PBA symptoms were clearly triggered by subjectively funny or upsetting/sad stimuli but were disproportionate in expression and were prolonged in duration. This is consistent with a model of PBA as a motor rather than non-motor phenomenon.

The impact of PBA was significantly correlated with the burden of symptomatic crying and not with that of laughing. This finding is perhaps not surprising and likely reflects the negative social reaction to public crying in comparison to that of laughing. The widely used CNS-LS attaches a greater weight to laughing episodes (20 points) than to crying (15 points). This discordance raises questions about the suitability of relying on total CNS-LS score as an outcome measure to evaluate the requirement for and response to treatment in routine clinical and therapeutic trial settings.

This study is not without limitations. No dedicated and domain-specific quality of life questionnaires were administered or semi-structured interviews conducted. The cross-sectional nature of this study precludes the longitudinal evaluation of the severity of PBA over time and the assessment whether symptoms plateau over time as sometimes observed clinically. Misdiagnosis of PBA was not specifically assessed in this study despite anecdotal evidence that PBA is commonly mistaken for psychiatric conditions. Yawning was also not specifically assessed despite the recognition that excessive yawning is a feature of PBA. Ideally, a non-MND neurological disease-control group such as PD, MSA, MS, vascular dementia or a post stroke group should have been included to contrast the prevalence and clinical characteristics of PBA in another neurological cohort. Antidepressant use was higher in the PBA group, albeit not significantly. This difference may reflect the use of anti-depressants to treat this symptom, and which has potential to impact scores in assessment of mood, anxiety and PBA itself. Anti-depressants were not stopped for this observational study and the impact of this treatment on reported symptoms in the study is unknown. A study in newly identified treatment naive patients with PLC would be informative. No patient was taking dextromethorphan/quinidine. Finally, the PLC-IQ, though based on patient interviews, has not yet been validated and cannot be taken to represent the full experience of patients with PBA. We also acknowledge the potential drawbacks of using the ALSFRS-r in this cohort. The ALSFRS-r is widely used in PLS studies [80–82], but the recently developed and validated PLS functional rating scale (PLSFRS) is a superior instrument to capture the full spectrum of motor disability associated with PLS and monitor progressive changes [83–86].

Notwithstanding these limitations, our data highlights that PBA is closely associated with pseudobulbar palsy and that is has a significant social and QoL impact. Our preliminary data demonstrate the practical ramifications of PBA, which, in light of the multitude of treatment options available, would justify routine screening for PBA and encourage early pharmacological intervention. Given the considerable clinical impact of PBA, large prospective studies should be conducted with the inclusion of multiple disease cohorts (PLS, MSA, MS, TBI etc.) to comprehensively assess both the aetiology and impact of this entity across various neurological conditions.

## Conclusions

This study supports the central role of pseudobulbar dysfunction in PBA. Furthermore, the continued use of the term PBA may serve as a reminder to clinicians, prompting urgent evaluation of wider pseudobulbar dysfunction and early interventions for dysarthria and dysphagia. This study provides support for classification of PBA in PLS as a pseudobulbar motor symptom strongly associated with other pseudobulbar features; particularly dysphagia, spastic dysarthria and brisk jaw-jerk. In contrast, PBA was not associated with non-motor features such as cognitive or behavioural impairment. PBA may therefore be conceptualised as "clonus" of emotion expression in which initially appropriate motor response is perpetuated through disinhibition of brainstem motor nuclei. Oppenheim’s hypothesis that PBA stems from the “interruption of the tracts which have an inhibitory effect upon bulbar centres” was, in his own words, “neglected by all the later writers, and has been put into the background”. In reference to the Jaw-jerk, Bickerstaff bemoaned that “this most helpful reflex is often ignored” [87]. Our study seems to provide support for both of these observations.

## Data Availability

Due to departmental policies, clinical, genetic or neuroimaging data from individual patients cannot be made available.
